# Subtle motor disturbances in PREDICT-PD participants

**DOI:** 10.1136/jnnp-2016-314524

**Published:** 2016-12-16

**Authors:** Alastair J Noyce, Anette Schrag, Joseph M Masters, Jonathan P Bestwick, Gavin Giovannoni, Andrew J Lees

**Affiliations:** 1 Department of Molecular Neuroscience, Reta Lila Weston Institute, UCL Institute of Neurology, London, UK; 2 Blizard Institute, Barts and the London School of Medicine and Dentistry, Queen Mary University of London, London, UK; 3 Department of Clinical Neuroscience, UCL Institute of Neurology, London, UK; 4 Wolfson Institute of Preventive Medicine, Queen Mary University of London, London, UK

## Abstract

**Objective:**

The PREDICT-PD study aims to identify increased risk of Parkinson’'s disease (PD) using online assessments of previously identified risk and early features of PD and an evidence-based scoring algorithm. We sought to determine whether higher risk participants (defined as those above the 15th centile of risk estimates) were more likely to have mild parkinsonian signs compared with lower risk participants.

**Methods:**

Video recordings of neurological examinations, including the Movement Disorder Society Unified Parkinson's Disease Rating Scale (MDS-UPDRS) part III, of 208 individuals who had previously completed an online risk assessment were scored blindly and independently by two movement-disorders experts. Higher risk and lower risk subjects were compared for MDS-UPDRS part III score (and derivations of this) to identify subclinical parkinsonism, and association of risk estimates with MDS-UPDRS III scores assessed.

**Results:**

Higher risk subjects had significantly higher median UPDRS part III scores (3, IQR 1–5.5) than lower risk subjects (1, IQR 0–3.0; p<0.001), and there was a significantly greater proportion of individuals classified as having subclinical parkinsonism. 18% of the higher risk subjects and 6% of the lower risk subjects exceeded the most stringent published cut-off for subtle parkinsonism of three definitions examined (p=0.027). Linear regression analysis demonstrated a continuous relationship of log-transformed risk estimates with UPDRS part III scores (increase in MDS-UPDRS per doubling of odds 0.52, 95% CI 0.31 to 0.72; p<0.001), which remained after adjustment for multiple vascular risk factors and scores on the Montreal Cognitive Assessment (0.58, 95% CI 0.30 to 0.87; p<0.001).

**Conclusions:**

The PREDICT-PD algorithm identifies a population with an increased rate of motor disturbances.

## Introduction

Despite significant advances in the field of Parkinson’'s disease (PD), it remains incurable and causes significant morbidity. The pathological process, leading to neuronal death in the substantia nigra pars compacta, begins years before motor disturbance causes functional handicap.^1^ Earlier detection could pave the way for clinical trials of treatments in individuals at greater risk of developing the disease.^2^

In the ‘prodromal phase’ or ‘prediagnostic phase’, there may be transient or subtle motor dysfunction, in parallel with non-motor features, prior to diagnosis.^3–8^ Subtle parkinsonian signs are common in older persons with an estimated prevalence of 30–40%.^9^ ^10^ While non-progression is a feature in some, their presence can herald cognitive decline or other adverse health outcomes, including frailty and death.^11^ ^12^

PREDICT-PD is a UK population-based cohort study which aims to identify individuals in the prediagnostic phase of PD using online assessments and an evidence-based algorithm. At baseline, PREDICT-PD recruited 1323 participants to complete online surveys, the answers to which yielded a combined risk estimate of PD. Participants were ranked from highest to lowest risk and a variety of comparative analyses undertaken between the extremes of risk.^13^ We here examined whether this approach can identify individuals with subtle motor features indicative of prodromal PD.

## Methods

The cohort was established in 2011 and the method of volunteer recruitment has been previously published.^13^ In brief, volunteers were individuals over the age of 60 years, without known neurological disease and residing in the UK. They completed an online consent form, questionnaire and keyboard-tapping test, and were sent the University of Pennsylvania Smell Identification Test (UPSIT) by post.^14^ ^15^ All aspects of the study were carried out in accordance with the Declaration of Helsinki and were approved by the Queen Square Ethics Committee (reference number 10/H0716/85). Baseline inclusion and exclusion criteria are presented in the online supplementary material.

10.1136/jnnp-2016-314524.supp1 supplementary material

### Online assessments

Every year participants answered an annual online survey and their risk of PD was estimated in accordance with responses. Estimates were based on previously identified factors, described in a systematic review and meta-analysis.^16^ Cross-sectional analyses have compared higher risk subjects (defined as those above the 15th centile of risk estimates), with lower risk subjects (participants with risk estimates below the 85th centile) in terms of objective smell and finger tapping, as well as subjective REM-sleep behaviour disorder (RBD).^13^ Detailed methods are described in the online supplementary material.

### Clinical examinations

From 2012, selected participants consented to a home visit, to determine whether the higher risk participants were more likely to have subtle motor features of PD compared with lower risk subjects. Participants were sampled with a preference for those estimated to be at the highest risk. Lower risk subjects (and middle risk) were required for comparative analysis and also to maintain blinding of participants to risk (for distribution of risk scores see online supplementary figure 1). Geographical location did not bias assessment and participants were seen regardless of where they resided in the UK. Acceptance rates to telephone invitations for home visits are provided in the online supplementary table S1.

During the domiciliary visit, a neurological examination was carried out by a trained clinician (AJN), including the Movement Disorder Society Unified Parkinson’'s Disease Rating Scale (MDS-UPDRS) motor section (part III), which was recorded on video (*all reference to MDS-UPDRS throughout the manuscript refers to part III motor scores only*).^17^ During the same visit, participants were assessed with the Montreal Cognitive Assessment (MoCA), which has been widely used as a cognitive screening tool.^18^

### Video scoring

Two senior movement disorders experts (AJL and AS), who were blind to risk estimates and were provided with no additional information about participants, independently scored the participant videos using the MDS-UPDRS.^17^ Rigidity scores were provided for each participant from the examination by AJN, given that this could not be ascertained from watching the videos. After all videos were scored, marks were compared between raters. Where no significant disparity existed between individual raters' scores (<5 point difference on the MDS-UPDRS), the scores from both were averaged. Where there was disparity in scores (≥5 points), the raters were asked to rescore again independently. If significant disparity remained, the raters rescored jointly to obtain a consensus score.

The original version of the UPDRS can be used to calculate an abbreviated score of ‘Mild Parkinsonian Signs’ (MPS). The most widely used version is that developed by Louis *et al*^10^ ^19^ (for details on scoring see online supplementary material). Bradykinesia does not feature in the MPS score, because its presence is an essential feature in the diagnostic criteria for PD.^20^ We therefore included bradykinesia as an additional outcome, considering bradykinesia as definite when combined scores from the MDS-UPDRS bradykinesia items (finger tapping, hand movements, hand pronation/supination, toe tapping and leg agility) were ≥1.5. We used a second definition of MPS or subthreshold parkinsonism described recently by an MDS Task Force, with scores >6 on the MDS-UPDRS (excluding action and postural tremor) indicating subthreshold parkinsonism.^3^

In addition to these two UPDRS-based scores, raters were asked to give a ‘global impression’ comment. After all videos had been rated, these comments were coded as the following ordered categorical variables: 0—normal, 1—minor clinical abnormality not necessarily associated with PD, 2—subtle clinical observation associated with PD, 3—suspected/possible early PD/parkinsonism, and 4—probable PD based on the Queen Square Brain Bank for Neurological Disorders diagnostic criteria.^20^ Again, these global impression scores were averaged for the two raters. Consensus was sought for ‘suspected’ or ‘probable’ PD (scores of 3 or 4). Cut-offs for our ‘global impression’ score were as follows: scores ≤1.0 considered as not indicative of parkinsonism, scores 1.5 to 2.5 indicative of subtle parkinsonism and ≥3.0 considered as clear evidence of parkinsonism.

### Statistical analysis

The aim of the study was to test the null hypothesis of no difference between the higher compared with lower (combined middle and lowest risk) risk subjects in the frequency and severity of motor dysfunction as assessed on the blinded ratings of the video recordings. Risk scores were calculated and higher risk and lower risk groups were calculated as described previously using the year 1 online assessment as this was closest to the home visits in the majority of cases.^13^

First, the scores from the raters were compared using intraclass correlation coefficients (ICC) and Bland-Altman plots, along with measures of bias and agreement. Where there was substantial disparity, measures were taken to obtain consensus before final scores were analysed (see above).

Second, the representativeness of sampled participants compared with the overall cohort was examined. Higher risk as well as lower risk participants that had been seen were compared with those not seen in terms of age, gender and other demographic information used for risk stratification, as well as MoCA scores, smell test scores and measures of motor function as described above.

Analyses using continuous and categorical motor outcomes were undertaken comparing higher risk subjects with lower risk subjects, using t-tests for parametric and Wilcoxon Rank Sum tests for non-parametric continuous data, Fisher's exact test for binary data, and the χ^2^ test for trend for ordinal data.

Linear regression was used to analyse the relationship of continuous risk estimates with MDS-UPDRS (excluding action and postural tremor) using the log odds of PD. Multivariate linear regression was undertaken to examine the influence of potential confounding factors such as prevalent cognitive impairment (using MoCA scores) and factors that increase risk of cerebrovascular disease. Vascular risk factors included: self-reported diabetes or use of antidiabetic drugs, hypertension or use of antihypertensives (ACE inhibitors, angiotensin receptor blockers (ARBs), calcium channel blockers (CCBs), β-blockers and diuretics), hypercholesterolaemia or use of statins, ischaemic heart disease and current smoking.

Age and gender were felt likely to confound the relationship between risk and motor outcomes, but their significant weighting in the algorithm meant that adjusting could lead to multicollinearity and increased standard error. Hence, a separate regression model was run in which the association between risk estimates without the contribution of age or gender and motor outcomes was examined to determine the role of other factors in the algorithm. All analyses were performed in Stata V.12 (StataCorp, College Station, Texas, USA).

## Results

Two hundred eight participants were assessed at home between October 2012 and September 2015. Four participants were excluded in line with the exclusion criteria of the study (see online supplementary material): one received lithium, two had cognitive impairment (MoCA <22) and one had had a previous stroke. Seven participants reported that they had been independently diagnosed with PD by neurologists during this period. All seven of these subjects were also reviewed in person, and identified as having PD by both raters on blinded video analysis. These were excluded from all further analyses. Video recordings were, therefore, scored and analysed from 197 participants.

The intraclass correlation coefficient (ICC) for scores by raters 1 and 2 was 0.78 (95% CI 0.71 to 0.84) after the first round. A difference between raters of ≥5 points on the MDS-UPDRS occurred for 24 out of 197 subjects (12%). These were rescored independently in round 2, resulting in an ICC of 0.86 (95% CI 0.81 to 0.89). After round 2, a difference between raters of ≥5 points still existed for 10 participants and these were scored jointly (final ICC 0.93; 95% CI 0.90 to 0.95). Measurements of agreement and bias, as well as graphs, from the Bland-Altman analysis are included in the online supplementary figure 2. Final consensus scores for MDS-UPDRS ranged from 0 to 19. The median global impression score was 0.5 (range 0–3.5) out of maximum of 4. Examples of videos and handwriting from participants with mild parkinsonism can be viewed in the supplementary material (see online supplementary figures 3–5 and videos 1–3; risk status and other information on these individuals is not disclosed).

10.1136/jnnp-2016-314524.supp2 supplementary video

10.1136/jnnp-2016-314524.supp3 supplementary video

10.1136/jnnp-2016-314524.supp4 supplementary video

Risk estimates from year 1 follow-up surveys were available for 185 participants. Median interval between the clinical assessment and online surveys was 95 (IQR 37–168) days. The median age of participants was 66.4 years (IQR 64.1–72.7); 94 were men and 91 were women. According to the pre-defined 15th centile risk estimate cut-off, 74 participants were deemed to be higher risk and the remaining 111 were lower risk (combined middle and lowest risk). Higher risk participants that were seen in-person had higher risk estimates and rankings, and were more likely to be male or have a first degree relative with PD, than the higher risk participants that were not seen, but in other aspects were comparable (see table 1). Lower risk participants that were seen in-person had lower risk estimates and rankings, and were younger and more likely to be current smokers than lower risk participants that were not seen, but in other aspects were comparable. There were no significant differences in intermediate outcomes (smell, subjective RBD and finger tapping) between participants that were seen and those that were not seen.

**Table 1:** Demographic, intermediate marker and risk information on higher and lower risk participants seen in person compared with those that were not seen

	Higher risk seen	Higher risk not seen	p Value		Lower risk seen	Lower risk not seen	p Value
n	74	75			111	770	
Age (95% CI)	72.2 (69.0 to 75.5)	70.8 (68.1 to 74.0)	0.252		64.9 (62.8 to 66.6)	67.1 (64.9 to 70.6)	<0.001
Male gender (%)	63 (85.1)	54 (72.0)	0.072		31 (27.9)	243 (31.6)	0.511
First-degree relative (%)	36 (48.6)	22 (29.3)	0.019		9 (8.1)	107 (13.9)	0.100
Current smoker (%)	1 (1.4)	0 (0)	0.497		11 (9.9)	17 (2.2)	<0.001
Past smoker (%)	38 (51.4)	31 (41.3)	0.252		51 (45.9)	293 (38.1)	0.119
Diabetes mellitus (%)	8 (10.8)	4 (5.3)	0.245		8 (7.2)	33 (4.3)	0.222
High cholesterol (%)	33 (44.6)	31 (41.3)	0.742		35 (31.5)	213 (27.7)	0.430
Ischaemic Heart disease (%)	13 (17.6)	11 (14.7)	0.662		3 (2.7)	31 (4)	0.791
Hypertension (%)	34 (45.9)	27 (36)	0.246		40 (36)	228 (29.6)	0.186
Drink coffee (%)	65 (87.8)	71 (94.7)	0.159		108 (97.3)	694 (90.1)	0.012
Drink alcohol (%)	67 (90.5)	66 (88)	0.792		101 (91)	674 (87.5)	0.351
Constipation (%)	22 (29.7)	29 (38.7)	0.301		17 (15.3)	113 (14.7)	0.776
Anxiety/depression (%)	14 (18.9)	15 (20)	0.999		13 (11.7)	87 (11.3)	0.873
Head injury (%)	36 (48.6)	36 (48)	0.999		26 (23.4)	218 (28.3)	0.309
Hyposmia ≤27/40 on UPSIT (109 available)	13 of 56 (23.2%)	16 of 53 (30.2%)	0.666	(653 available)	8 of 88 (9.1%)	76 of 565 (13.3%)	0.307
Subjective RBD ≥5 on RBDSQ (147 available)	18 of 75 (24.0%)	10 of 72 (13.8%)	0.141	(880 available)	14 of 112 (12.5%)	81 of 768 (10.5%)	0.513
Slow finger tapping KS ≤44 taps in 30 s (124 available)	21 of 67 (31.3%)	12 of 57 (21.1%)	0.161	(749 available)	12 of 92 (13.0%)	100 of 657 (15.2%)	0.754
Risk estimate year 1 (IQR)	9.9 (6.9–15.1)	15.3 (12.3–18.2)	<0.001		136.8 (54.3–207.3)	78.1 (48.2–118.7)	<0.001
Rank year 1 (IQR)	54 (25–100)	103 (71–133)	<0.001		860 (424–1001)	580 (373–784)	<0.001

Information from survey answers in year 1. Hypertension defined according to self-report or use of diuretics, ACE inhibitors and angiotensin receptor blocking drugs, calcium channel blockers, and beta blockers (including propranolol, atenolol, bisoprolol, but not sotalol or timolol). High cholesterol defined according to self-report or statin use. Diabetes mellitus defined according to self-report or use of metformin, gliclazide or insulin.

UPSIT, University of Pennsylvania Smell Identification Test; RBDSQ, RBD screening questionnaire; KS, kinesia score.

### Comparison of higher and lower risk groups

Higher risk participants were significantly more likely to have motor impairment on the MDS-UPDRS and all derived scores for MPS, compared with lower risk (see table 2). The median MDS-UPDRS score in higher risk subjects was 3 (IQR 1.0–5.5) and in lower risk subjects was 1 (IQR 0.0–3.0; p<0.001). According to the definition of subthreshold parkinsonism by the MDS Task Force (MDS-UPDRS >6 not including kinetic tremor and postural tremor^3^), 13/74 (17.6%) higher risk compared with 7/111 (6.3%) lower risk met the cut-off (p=0.027). For MPS cut-offs (also derived from UPDRS) proposed by Louis *et al*,^19^ the proportion was 31.1% higher risk and 10.8% lower risk (p=0.001).

**Table 2:** Comparison of total motor MDS-UPDRS scores and MoCA scores, and proportion of participants meeting three definitions of mild parkinsonian signs, between higher and lower risk participants

	Higher risk	Lower risk	p Value
n	74	111	
Median MDS-UPDRS (IQR)	3 (1.0–5.5)	1 (0.0–3.0)	<0.001
Mild parkinsonism Berg definition n (%)	13 (17.6%)	7 (6.3%)	0.027
Mild parkinsonism Louis definition n (%)	23 (31.1%)	12 (10.8%)	0.001
Global impression n (%)			
0–1.0	55 (74.3%)	103 (92.8%)	
1.5–2.5	17 (23.0%)	7 (6.3%)	0.001
3+	2 (2.7%)	1 (0.9%)	
Median MoCA (IQR)	27 (26–28)	28 (26–29)	0.049

MDS-UPDRS, Movements Disorders Society Unified Parkinson's Disease Rating Scale; MoCA, Montreal Cognitive Assessment.

Using the ‘global impression’ score, three participants were suspected to have undiagnosed PD (scores ≥3 occurred in 2.7% of higher risk vs 0.9% of lower risk). Once they were excluded from this specific analysis, 23.0% of higher risk subjects were suspected to have subtle features of parkinsonism (scores ≥1.5 and <3) compared with 6.3% of lower risk (p=0.001). Bradykinesia (score ≥1.5 taking all body regions into account) was present in 22/74 (29.7%) of higher risk, compared with 17/111 (15.3%) of lower risk (p=0.019). MoCA scores were slightly lower in higher risk compared with lower risk participants, with 27 (IQR 26–28) and 28 (IQR 26–29) respectively (p=0.049). Finally, we divided the lower risk group again into middle risk and lowest risk (according to the 85th centile risk estimate), giving rise to 58 and 53 participants in each group respectively. The above analyses were repeated using the χ^2^ test for trend and confirmation of a gradient across risk groups was observed for each MPS score (see online supplementary table S2).

### Association of risk estimates and MDS-UPDRS scores

In regression analysis, risk scores were significantly associated with MDS-UPDRS score (excluding action and postural tremor as per MDS Task Force Criteria^3^). The increase in MDS-UPDRS per doubling of risk (expressed as an odds) was 0.52 points (95% CI 0.31 to 0.72; p<0.001; see table 3). Associations adjusted for multiple cerebrovascular disease risk factors remained statistically significant (increase in MDS-UPDRS 0.52 points; 95% CI 0.30 to 0.74; p<0.001), as were associations after adjustment for MoCA scores (0.56 points; 95% CI 0.30 to 0.83), and after adjustment for both together (0.58 points; 95% CI 0.30 to 0.87). Risk scores that excluded age and gender (crude and adjusted for vascular risk factors and MoCA scores) were also strongly associated with MDS-UPDRS score (p<0.001 for all analyses).

**Table 3:** Regression analysis for the association between risk estimates and motor MDS-UPDRS scores (crude and adjusted)

Crude exposure	Adjusted for	Increase in MDS-UPDRS per doubling of odds	95% CI	p Value
Log odds		0.52	0.31 to 0.72	<0.001
	All vascular*	0.52	0.30 to 0.74	<0.001
	MoCA	0.56	0.30 to 0.83	<0.001
	All vascular* and MoCA	0.58	0.30 to 0.87	<0.001
Log odds minus age and gender		0.51	0.30 to 0.72	<0.001
	All vascular*	0.54	0.31 to 0.76	<0.001
	MoCA	0.55	0.28 to 0.82	<0.001
	All vascular* and MoCA	0.59	0.30 to 0.88	<0.001

*Vascular factors include diabetes mellitus, high cholesterol, heart disease, hypertension, smoking.

MoCA, Montreal Cognitive Assessment.

## Discussion

MPS may be observed in up to 40% of community dwelling older persons and are associated with a range of negative health outcomes. Analogous to mild cognitive impairment and dementia, the features and determinants of deterioration from ‘mild’ to ‘clinically relevant’ impairment are poorly defined, and most unselected older people with MPS will not be subsequently diagnosed with PD.^21^

Diagnosis is made when patients present to their doctor with a constellation of physical signs that reliably predict the characteristic pathological findings at postmortem (severe pars compacta nigral cell loss with Lewy bodies).^20^ In retrospect many patients and relatives report that subtle motor impairment may have been present for several years before medical attention was sought.^5–7^ Given that Lewy bodies are found in about 10% of brains of older individuals without a diagnosis of PD at postmortem, it is likely that a substantial proportion of individuals with MPS will however have been in the early stages of PD.^20^

We have previously shown that older members of the UK population, without any known neurological disease, could be stratified online for future risk of PD as suggested by ‘intermediate markers’ of poorer smell and finger tapping, using objective tests, and greater subjective RBD, as well as an increased risk of future diagnosis of PD.^13^ ^16^ ^22^ We here present data that demonstrate that this methodology also identifies individuals with MPS. MPS were defined using a range of scoring methods with varying stringency. Higher risk subjects were significantly more likely to have subtle motor signs, with higher MDS-UPDRS scores, and a greater proportion exceeded cut-offs for MPS defined by the MDS Task Force, the MPS score by Louis *et al*, and our own ‘global impression’ score.^3^ ^19^ The higher proportion of participants with MPS according to the definition by Louis *et al*^10^ ^12^ compared with the other criteria may be in part due to our use of the MDS-UPDRS for scoring as opposed to the original UPDRS (see methods and online supplementary materials). The MDS-UPDRS is more sensitive to subtle changes in movement, particularly those relating to repetitive movement such as finger tapping. We also observed a greater proportion of higher risk subjects (30%) with bradykinesia compared with lower risk (15%).

MPS have been associated with increasing age, cognitive impairment and cerebrovascular disease.^21^ The strength of association between estimated risk of PD and MDS-UPDRS in our study tended to increase when age and gender were dropped from the analysis and when analyses were adjusted for a combination of vascular risk factors and MoCA scores, suggesting that while age and gender are included in the algorithm, neither these nor vascular risk factors nor mild cognitive impairment are sufficient to account for the association. Together these results, given the strength of associations and the consistency across criteria using blinded assessments, suggest that at least in some higher risk subjects these MPS are a harbinger for future PD rather than simply a form fruste or mimic driven by vascular disease or mixed pathology.

Although seven patients with PD diagnosed independently during study follow-up were excluded from the outset, the ‘global impression’ score and associated MDS-UPDRS scores for three additional cases suggested that they may have been prevalent cases of undiagnosed PD. Excluding these three subjects did not alter any of the results (*note these are not the participants depicted in the videos)*.

Other groups have identified MPS in volunteers with individual risk factors for PD. In the Bruneck Study Cohort, increasing age, vascular risk factors, loss of olfactory function and hyperechogenicity in the region of the substantia nigra using transcranial sonography were found to be predictive of MPS.^23^ In the TREND study, olfactory deficit, subjective RBD, male gender and nigral hyperechogenicity were linked to MPS.^24^ Shulman *et al*,^25^ used genotyping data to explore associations between PD risk loci and MPS, finding associations between *MAPT* and *CCDC62* with global parkinsonism and bradykinesia specifically. Separately, an analysis of a large series of volunteers without a diagnosis of PD found an association between MPS elicited during in life and neuronal loss in the nigra at postmortem, but not Lewy body pathology, raising the possibility that neuronal loss underlies these signs.^26^ Finally, Postuma *et al*,^8^ prospectively demonstrated the gradual emergence of objective and rating scale-derived features of PD in a cohort of participants with idiopathic RBD, up to the point at which a proportion met diagnostic criteria for parkinsonism. This study nicely showed how MPS can evolve over time, but RBD represents a specific model for prediagnostic PD, which may not be the same as the course that most PD patients follow. To the best of our knowledge, this is the first time a composite PD risk score has been used to identify probable MPS at the population level.

The magnitude and consistency of associations makes chance an unlikely explanation for the results but as with other aspects of the PREDICT-PD study, selection bias may have played a part and we acknowledge that the proportion of subjects with a family history of PD is higher than in the general population. However, the frequency of a positive family history is not as high as reported in the PARS study, and is similar to the TREND study, despite the investigators more stringent definitions.^27^ ^28^ Furthermore, while we excluded subjects with an incident diagnosis of PD during follow-up, we cannot discount that some of the participants may have taken part because they suspected that they might have early signs of PD. Observer bias would have been substantially reduced by blinding raters to participant risk estimates. Good ICCs were obtained between the two raters for participants included in the analysis, despite the great difficulty in rating subtle features compared with well-established parkinsonian features, and were excellent after contentious cases were reviewed again. Likewise, agreement between raters was also acceptable in Bland-Altman analyses. ICCs were substantially better when PD cases were included, which emphasises the fact that subtle parkinsonism can be hard to detect (see online supplementary material). Finally, this cross-sectional analysis of MPS may have been prone to measurement error given recent evidence that MPS may be transient in the early stages of PD.^29^

Taken together with earlier work showing an increase of intermediate markers for PD (olfactory dysfunction, subjective RBD, and slow finger tapping) in the higher risk group, we have increasingly strong evidence to support enrichment for underlying risk of PD in PREDICT-PD.^13^ Future work in these participants will seek to define the course of subtle parkinsonian signs, their relationship with other symptoms in the prediagnostic phase and those features that best predict future diagnosis of PD.
