# Supplementary material for: Subtle motor disturbances in PREDICT-PD participants
Source: J Neurol Neurosurg Psychiatry. 2016 Dec 16;88(3):212–7. doi: 10.1136/jnnp-2016-314524 (PMC5529958; doi:10.1136/jnnp-2016-314524)
Supplement: supplementary material [file jnnp-2016-314524supp001.pdf]

SUPPLEMENTARY MATERIAL

Baseline inclusion criteria

1. Residency in the UK
2. Age 60–80 years

Baseline exclusion criteria

1. Pre-existing PD, movement disorder, stroke, motor neuron disease, dementia
2. Drug usage known to be associated with iatrogenic parkinsonism

Further information about online assessments and risk scoring

Since 2011, participants have answered an annual online survey and their risk of PD was estimated each year using an algorithm and in accordance with their responses. Estimates were based on previously identified factors, described in a systematic review and meta-analysis.[16]

The algorithm included age, gender, smoking status, first degree relative with PD, coffee use, alcohol use, hypertension, NSAID use, calcium channel blocker use, beta blocker use, constipation, previous head injury, anxiety or depression and erectile dysfunction (in males only). Most factors were sought in binary terms (i.e. presence or absence) except for bowel movement frequency (7 possible answers for frequency with a cut off of less than 1 movement per day denoting low frequency or laxative use), erectile dysfunction (3 options with 'poor' indicating dysfunction) and mood (a cut off score of 11 or above in either the anxiety or depression components of the HADS questionnaire denoting moderate forms of these disorders or antidepressant use). In order to keep the survey simple, pesticide exposures, proxies for organo-chemical exposure, and more complicated factors were not included.

Following each annual review, volunteers carried out a keyboard-tapping test (the Bradykinesia Akinesia Incoordination; BRAIN-tap test) with each hand.[14] The most

useful parameter that the BRAIN-tap test generates is the kinesia score (or KS), which is the number of alternate taps in 30 seconds. At baseline and year 3 of follow-up, participants also completed the University of Pennsylvania Smell Identification Test (UPSIT, US version).[15] Cross-sectional analyses have compared higher risk subjects (defined as those above the 15th centile of risk estimates), with lower risk subjects (participants with risk estimates below the 85th centile) in terms of objective smell and finger tapping, using the instruments described above, as well as subjective REM-sleep behaviour disorder (RBD) assessed using a validated questionnaire.[13]

Table S1: Participants contacted for home visit in the cohort

	Higher risk	Middle risk	Lower risk	Total
n	198	927	198	1323
Not approached	85 (43%)	870 (94%)	107 (54%)	1062 (80%)
Seen	89 (45%)	50 (5%)	69 (35%)	208 (16%)
Approached, declined	4 (2%)	0	4 (2%)	8 (0.6%)
Unable to see	3 (2%)	3 (0.3%)	10 (5%)	16 (1%)
Not contactable	17 (9%)	4 (0.4%)	8 (4%)	29 (2%)

Legend: Breakdown of responses from participants that were contact by telephone to be invited to participate in a home visit.

Figure 1: Histogram demonstrating the distribution of risk scores in the higher and lower risk groups

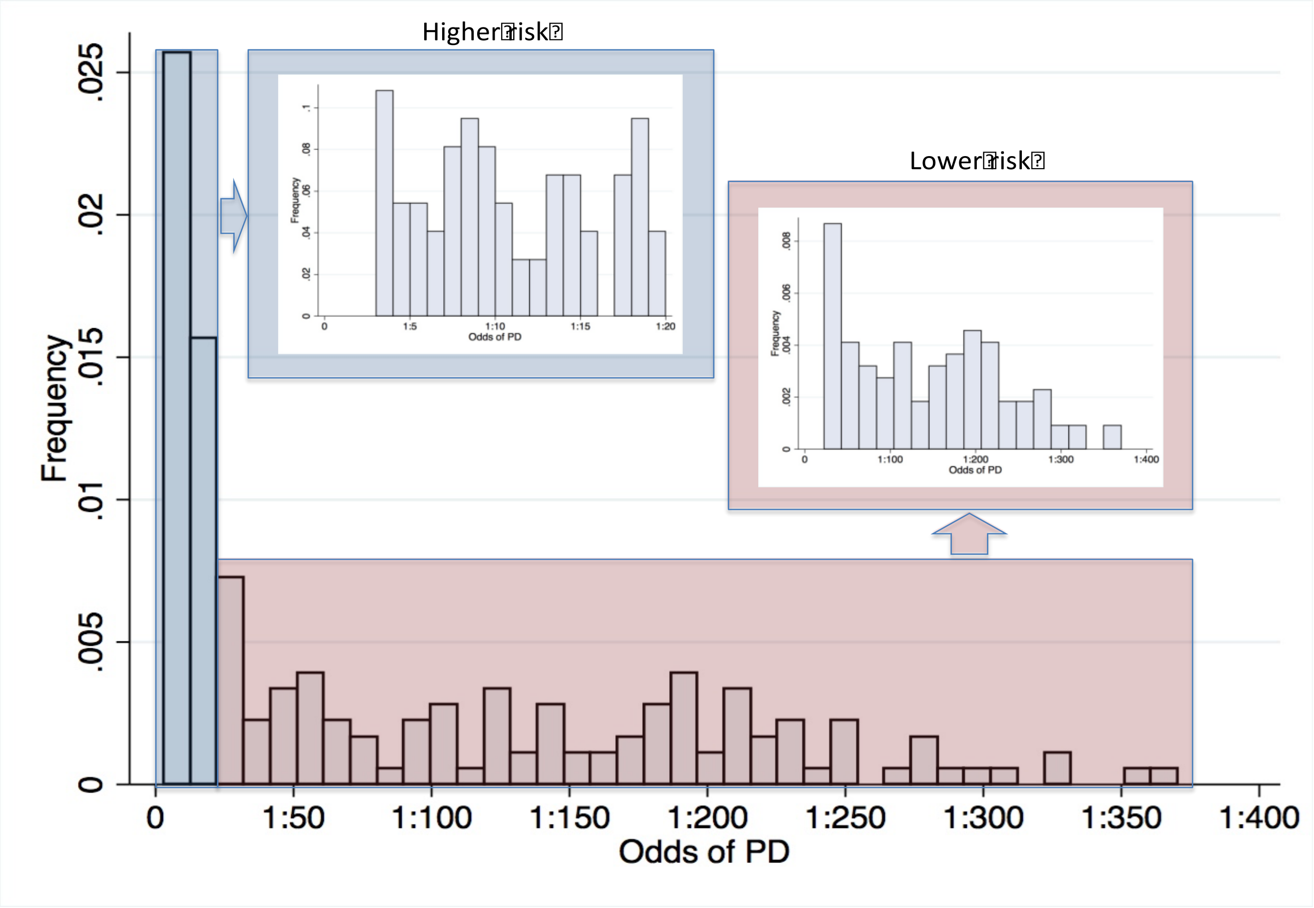

Legend: Sampling was performed with a preference for the highest risk participants and aiming for a balanced distribution across remaining risk estimates.

Further information on scoring Mild Parkinsonian Signs

The most widely used version is that developed by Louis and colleagues.[10,19] The MPS score was derived from ten items from the UPDRS including facial expression, speech, tremor at rest (in any body region), rigidity (rated separately at the neck, right arm, left arm, right leg and left leg), posture and body bradykinesia. In the present study, MPS were defined according to a binary definition (i.e. present or absent) when any of the following conditions was met: 1) two or more UPDRS ratings = 1, or 2) one UPDRS rating ≥2, or 3) UPDRS rest tremor rating ≥1.[19] The MDS-UPDRS was designed for raters to 'rate what they see' and was used in this study instead of the older version of the UPDRS. Differences between the two scales relate mainly to subtleties in repetitive movements, which do not form part of the MPS score defined here. Therefore we used the MDS-UPDRS to apply the MPS criteria acknowledging that there may be slight over diagnosis of MPS when using this instead of the UPDRS, but any differences would likely be slight.

Inter-rater Reliability and Agreement

Intra-class correlation coefficient (ICC) for scores by raters 1 and 2, without exclusion of participants with incident PD (n=7) was 0.90 (95% CI 0.87 to 0.92) after the first round, 0.93 (95% CI 0.91 to 0.95) after round 2 and, 0.97 (95% CI 0.96 to 0.98) after the consensus round. The improvement in ICC with PD patients included, compared to when they were excluded (as presented in the main body of the manuscript), emphasises the difficulty in rating subtle parkinsonism in those without overt signs.

Bias and Agreement from Bland-Altman Analysis

After round 1 of scoring, the median UPDRS score from rater 1 (AJL) was 2.0 (IQR 1.0-5.0) and for rater 2 (AS) was 1.0 (IQR 0.0-4.0). There was bias of 1.12 points in

favour of towards rater 1 (SD 2.84), with 95% limits of agreement of -4.45 to 6.70. After round 2 of scoring, bias was reduced marginally to 1.10 points (SD 2.39), with 95% limits of agreement of -3.58 to 5.79. After the consensus round of scoring, bias was 0.85 points towards rater 1 (SD 1.67) with 95% limits of agreement of -2.43 to 4.13 (see Figure 2 below).

Figure 2: Bland-Altman plots depicting inter-rater agreement with difference in MDS-UPDRS scores plotted against average.

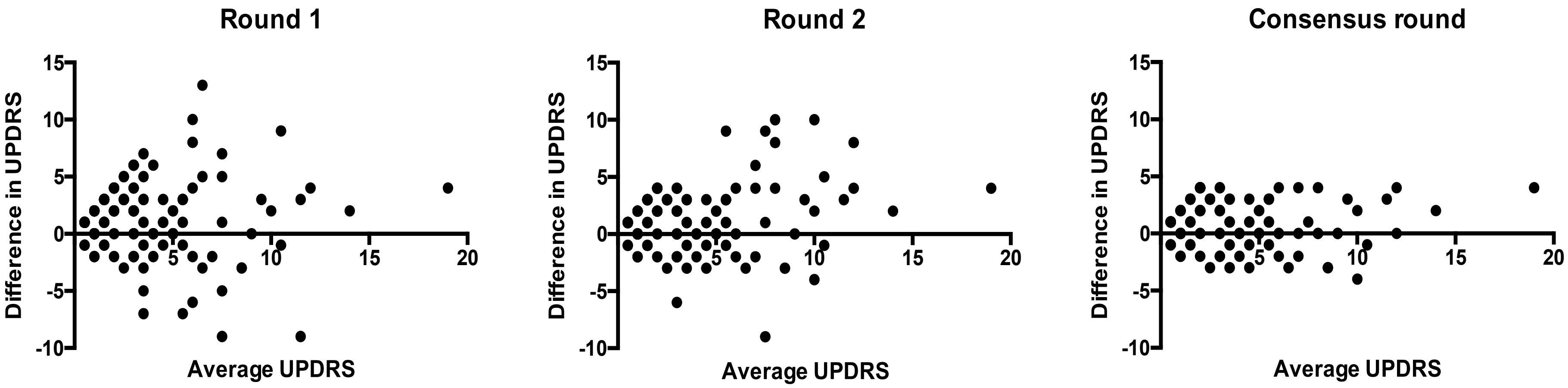

Legend: Bland-Altman plots comparing rater scores (difference in scores plotted against the average of the scores) after round 1, round 2 and the consensus round of scoring.

Video Legend

Participant 1: the participant in this video displays mild facial hypomimia, minor involuntary movement in the left hand at rest, impaired finger tapping in the left upper limb, mild right upper limb rigidity and reduced arm swing.

Participant 2: the participant in this video displays a breakdown in right finger tapping. She walks with reduced right arm swing and holds the arm slightly flexed.

Participant 3: the participant in this video displays facial hypomimia, breakdown of repetitive movement in the left upper limb and reduced arm swing. Foot tapping is also slow, with the left more so than right.

Figure 3: Example of handwriting from participant 1

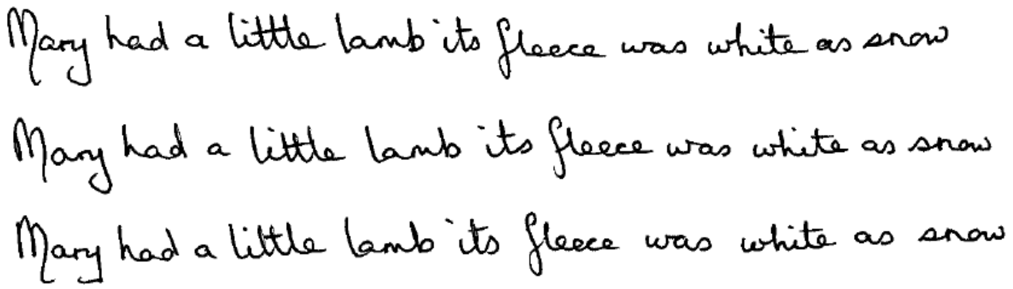

Legend: no clear evidence of micrographia, but subtle variation in word spacing horizontally and vertically.

Figure 4: Example of handwriting from participant 2

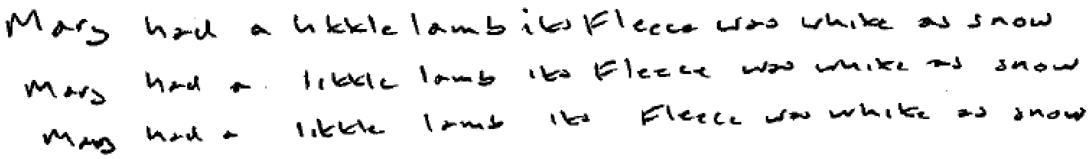

Legend: clear micrographia and decrement in sentence length.

Figure 5: Example of handwriting from participant 3

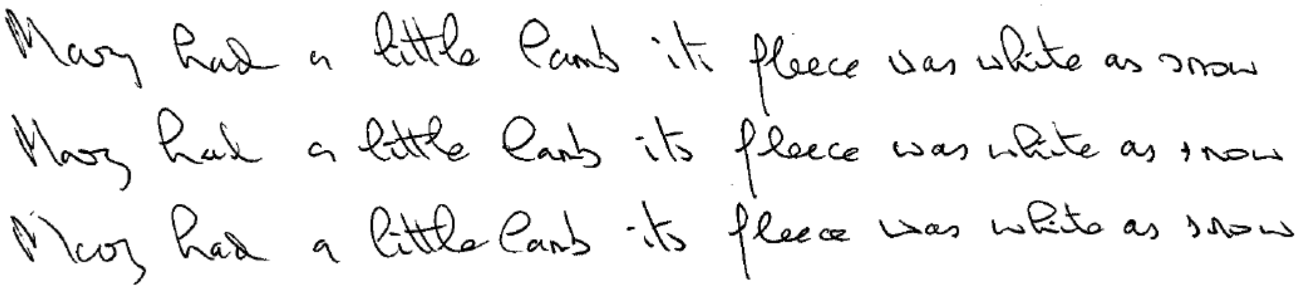

Legend: unremarkable handwriting.

Table S2: Comparison of total motor MDS-UPDRS scores and proportion of participants meeting three definitions of mild parkinsonian signs, between higher, middle and lower risk participants

	**Higher risk**	**Middle Risk**	**Lower risk**	**p-value***
n	74	58	53	
Median MDS-UPDRS (IQR)	3 (1.0-5.5)	2 (0.5-3.0)	0.5 (0.0-2.0)	
Mild parkinsonism Berg definition n (%)	13 (17.6%)	4 (6.9%)	3 (5.7%)	0.053
Mild parkinsonism Louis definition n (%)	23 (31.1%)	8 (13.8%)	4 (7.6%)	0.002
Global impression n (%)				
- 0–1.0	55 (74.3%)	52 (89.7%)	51 (96.2%)	
- 1.5–2.5	17 (23.0%)	5 (8.6%)	2 (3.8%)	0.003
- 3+	2 (2.7%)	1 (1.2%)	0 (0%)	

Legend: MDS-UPDRS = Movements Disorders Society Unified Parkinson's Disease Rating Scale; MoCA = Montreal Cognitive Assessment; IQR = interquartile range; * Chi-square test for trend.
